# Supplementary material for: High-energy photoemission final states beyond the free-electron approximation
Source: Nat Commun. 2023 Aug 10;14:4827. doi: 10.1038/s41467-023-40432-5 (PMC10415355; doi:10.1038/s41467-023-40432-5)
Supplement: Supplementary file 1 — Supplementary Information [file 41467_2023_40432_MOESM1_ESM.pdf]

Supplementary Information

1. KKR calculations with multiple-scattering final states

1.1 Computational scheme

In the first step of our theoretical investigations, we performed self-consistent electronic structure calculations within the ab-initio framework of the spin-density functional theory in order to generate the self-consistent-field (SCF) potential for further photoemission calculations. The LDA potential of Vosko et al. was used [1]. The electronic structure of semi-infinite crystal was calculated within the relativistic multiple scattering approach using the Green's function Korringa-Kohn-Rostoker (KKR) formalism in the tight binding mode [2]. The experimental lattice constant (a = 4.09 Å) was used. In order to achieve precise description of the most subtle details of the SCF potential, important for photoemission at high excitation energies, the multipole expansion of the Green's function employed an unusually large angular-momentum cutoff l_{max} of 5. In addition, a large number of **k**-points (36x36x36) in the first surface BZ was used. The self-consistent calculations have been performed in two modi, within the so-called atomic sphere approximation (ASA) and in the full potential (FP) mode.

The obtained SCF potential was used for the photoemission calculations within the one-step model. The final states (the time-reversed LEED state) were treated using the so-called layer KKR technique [3], allowing accurate description of these states in a wide $h\nu$ range starting from 6 eV up to several keV. To ensure the convergence of the multiple scattering between the layers, our calculations used a plane-wave basis where the number of the surface reciprocal lattice vectors **g** was increased to 147 instead of the default value 37 [2]. Another important ingredient of the multiple-scattering calculations is an accurate description of the kinematic and dynamic effects in both initial and final states. For the latter, the dynamic effects are taken into account via the X-matrix [4] which represents the energy-dependent multiple scattering within a single layer. Whereas in VUV-ARPES the kinematic and dynamic effects are comparable, in the soft- and hard-X-ray regime the dynamic effects weaken, whereby the X-matrix approaches zero, leading to the so-called single-site scattering approximation. Another important parameter in the description of multiple scattering is connected with the expansion of all physical quantities in terms of angular momentum l, i.e. using the Bauer's identity to represent plane waves (scattering between the layers) and spherical waves (inside the layer). These expansions involve a summation over l that must be truncated at a certain value l_{max}. In this context, the increase of l_{max} should be viewed rather as an extension of the basis set for accurate description of the multiple scattering than physically meaningful l-channels in the scattering process. A simple assessment of l_{max} can be obtained from the radial Schrödinger equation where, in order to scatter on the spherical potential, the electron must first overcome the centrifugal barrier $l(l+1)/a^2$ (a is the atomic radius). This implies only the partial waves, whose l satisfies the inequality $k^2 > l(l+1)/a^2$, should be included into the l-expansion. The higher E_k, the larger l_{max} needs to be used (for the detailed explanation see [5]). For E_k in the range 300-1300eV, considered here, l_{max} falls between 4 and 5. The calculations have been performed for a finite temperature of 20K leading to an additional final-state **k**-broadening, increasing with $h\nu$ [6].

1.2 Effect of various approximations for the multiple-scattering process

We have made an effort to elaborate our ARPES calculations towards their quantitative agreement with the experiment in a few successive steps:

– Supplementary Figure 1 shows the results obtained with multiple-scattering final states, as opposed to the FE final states used for the calculations in Figs. 1 and 2 in the main text, under successive refinements of the computational approximations:

– The results in Supplementary Figure 1(a) were obtained within the ASA and l_{max} = 3. Due to the non-FE effects described by the multiple-scattering final states, they already show spectral structures due to the MBFSs (such as where marked by magenta arrows) although mostly on the low-energy end of our *hv* range and not exactly in the same **k**-space regions compared to the experiment;

– The inclusion of warping of the potential in the interstitial and surface regions within the FP scheme [7],Supplementary Figure 1(b), does not result in any significant improvement in our case of Ag. Nevertheless, we anticipate that the accurate FP will be crucial for more open crystal structures, covalent materials, van-der-Waals materials, etc. where the potential modulations are sharper [7]. As we have seen in the present work, their accurate description should be particularly important at high E_k where the final states are highly sensitive to the high-frequency modulations of $V(\mathbf{r})$ and thus to the accurate representation of its real-space variations;

– Another step, the inclusion of the full X-matrix compared to the single-site approximation, presented in Supplementary Figure 1(c), considerably improves the description of the relative intensity variations in the *hv* interval between 400 and 500 eV (magenta arrow, for example) but does not notably affect the spectral intensity at higher energies. This observation can be understood from analysis of scattering amplitude $f_k(\theta)$, giving more insight into the scattering process. The calculations of $f_k(\theta)$ for Ag by Sébilleau et al. [8], reproduced in Supplementary Figure 2, demonstrate that for E_k above ~400 eV it is strongly dominated by forward scattering. In practice, this means that for these energies the electrons scatter essentially along the rows of atoms, justifying the single-site approximation for the multiple scattering;

– Finally, at the last step of our computational refinement presented in the Supplementary Figure 1(d), we increased l_{max} from 3 to 5. As expected, not only has this returned a vivid pattern of the MBFS-induced replica bands and added spectral broadening at low E_k (such as where marked by magenta and yellow arrows, respectively) but also pushed these effects to yet higher *hv* up to 700 eV (magenta arrow). Further increase of l_{max} would inflate the computational time beyond presently realistic.
Although these successive refinements of the computational scheme do move towards a better description of the experiment, the achieved agreement with the experimental results can only be considered as qualitative regarding both dispersions and intensities of the ARPES structures. We conjecture that the remnant deviation may trace back to quite small sensitivity of the total energy to high-frequency components of the crystal potential. Therefore, the total-energy minimization used to generate the self-consistent potential in the DFT calculations may not ensure sufficient accuracy of its high-frequency components which critically affect the hybridization and thus non-FE effects in the final states at high energies. The accuracy of the final states used in the ARPES calculations can in principle be verified independently from the initial states by calculating the LEED spectra and their fitting to the experiment using the methodology previously developed for very low energies (see [9–11] and the references therein). In any case, including the subtle details of $V(\mathbf{r})$ within the FP approach and the use of sufficiently large l_{max} give the best possible single-particle description of the photoemission final state.

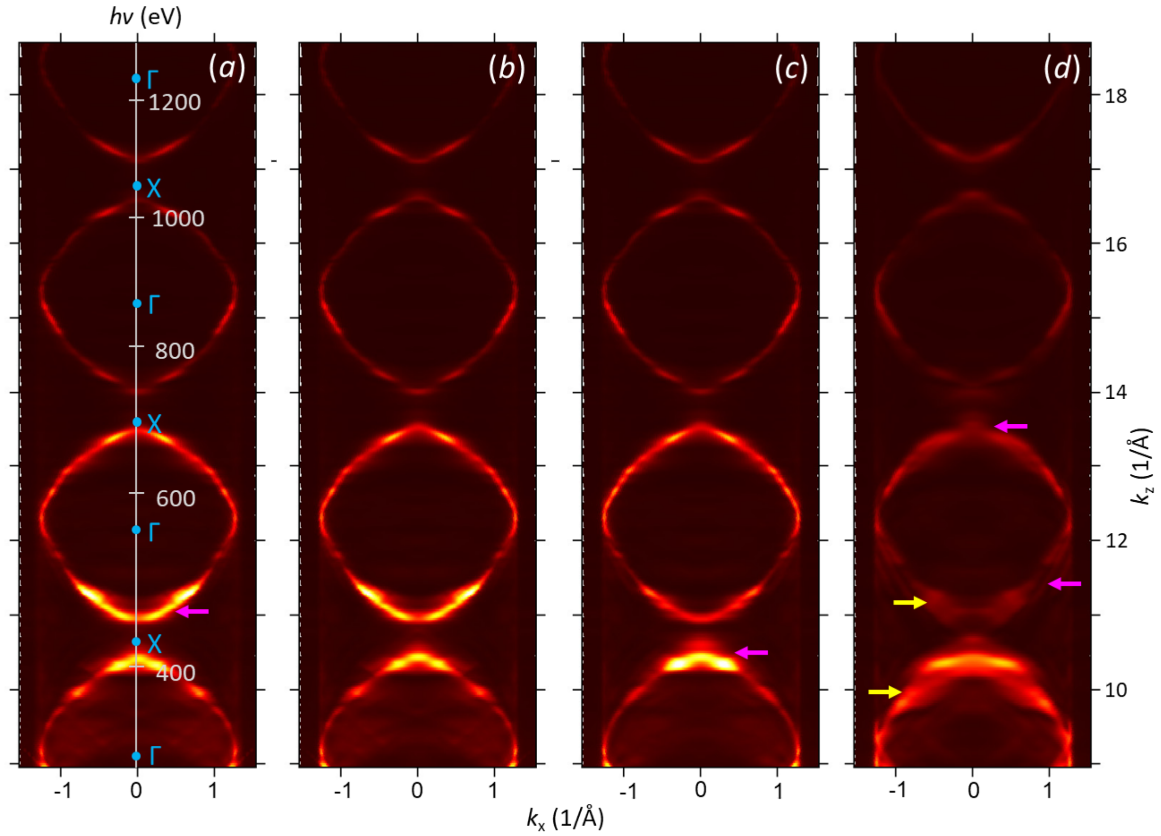

Supplementary Figure 1. One-step ARPES calculations as in Fig. 1(d) but using multiple-scattering final states under successive refinements of their treatment: (a) standard spherical-wave expansion and single-site scattering approximation; (b) adding full potential; (c) the full X-matrix beyond the single-site scattering;(d) increasing the angular momentum expansion to l_{max} = 5. The calculations reproduce the multiple spectral peaks (magenta arrows) and the added spectral broadening (yellow) induced by the MBFSs.

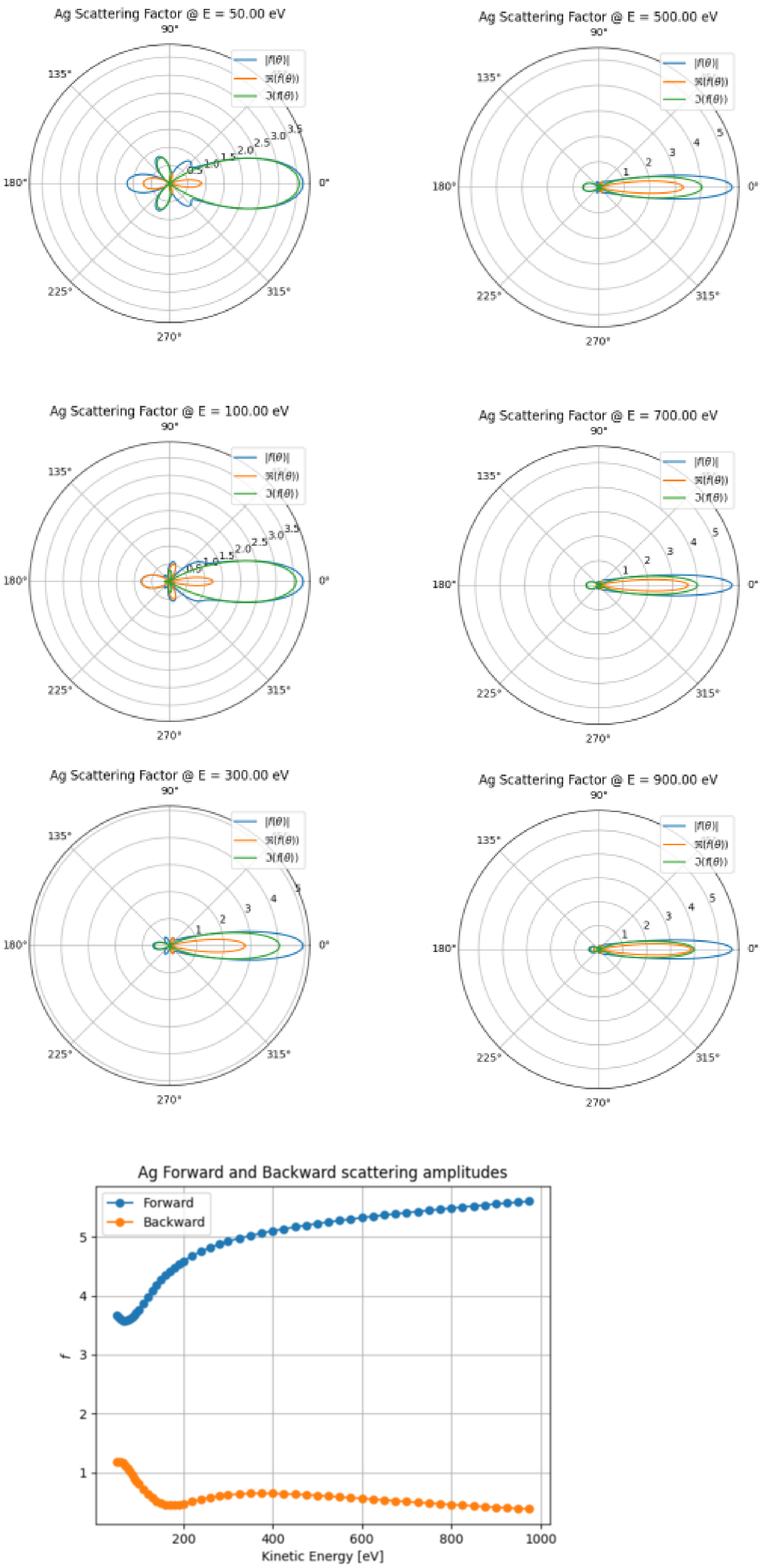

Supplementary Figure 2. Scattering amplitude $f_k(\theta)$ for Ag as a function of E_k (diagram panels on the top) and the total forward and backward scattering contributions (bottom panel).

2. Matching formalism in LEED: Complex band structure, wavefunction matching, connection to individual final-state bands

The matching approach of the LEED dynamic theory is based on matching of the electron wavefunction in the vacuum half-space (superposition of the incident and all diffracted plane waves) to that in the crystal half-space (superposition of Bloch waves decaying into the crystal interior). The core of the matching approach is the calculation of complex band structure as a function complex surface-perpendicular momentum. The most profound advantage of the matching approach is tracing the direct connection of the LEED and ARPES spectra to the final-state band structure. Here, we outline the main computational details of the matching approach.

2.1 Complex band structure of semi-infinite crystal

Underlying the LEED calculations within the matching approach is the complex band structure *E*(**k**) of the crystal terminated by the surface (semi-infinite crystal). Whereas in the infinite crystals the translation invariance allows only propagating Bloch waves $\phi_\mathbf{k}$ with real **k**, in the semi-infinite one the solutions of the Schrödinger equation are $\phi_\mathbf{k}$ decaying into the crystal interior with complex $k_\perp$. Calculations of the corresponding complex *E*(**k**) with the absorption potential V_i is a way more difficult compared to usual band structure calculations with real **k** [12-16].

Here, we will outline calculations of the complex *E*(**k**) based on the simplest empirical pseudopotential scheme. In this case $\phi_\mathbf{k}$ are calculated as a plane-wave expansion $\phi_\mathbf{k} = \sum_\mathbf{G} C_\mathbf{G}\, exp(i(\mathbf{k}+\mathbf{G})\mathbf{r})$, where **G** are the bulk reciprocal-lattice vectors, which reduces the stationary Schrödinger equation $\left(-\frac{\hbar^2}{2m}\nabla^2 + V - iV_i - E\right)\phi_\mathbf{k} = 0$ with the pseudopotential ***V*** to a homogeneous system of equations

$$\sum_\mathbf{G}\left(\frac{\hbar^2}{2m}|\mathbf{k}+\mathbf{G}|^2\delta_{\mathbf{GG'}} + V_{\mathbf{GG'}} - iV_i\delta_{\mathbf{GG'}} - E\delta_{\mathbf{GG'}}\right)C_\mathbf{G} = 0,$$

where $V_{\mathbf{GG'}}$ are the matrix elements $V_{\mathbf{GG'}} = \int exp(-i(\mathbf{k}+\mathbf{G})\mathbf{r})V\, exp(i(\mathbf{k}+\mathbf{G'})\mathbf{r})d\mathbf{r}$ (for the local ***V*** = *V*(**r**) equal to its Fourier components $\int V(\mathbf{r})e^{i(\mathbf{G'}-\mathbf{G})\mathbf{r}}d\mathbf{r}$). This system can be solved under the condition

$$\det\left(\left\{\frac{\hbar^2}{2m}|\mathbf{k}+\mathbf{G}|^2\delta_{\mathbf{GG'}} + V_{\mathbf{GG'}} - iV_i\delta_{\mathbf{GG'}} - E\delta_{\mathbf{GG'}}\right\}\right) = 0$$

which is the secular equation, connecting *E* и **k** in the complex *E*(**k**). For the infinite crystal (implying V_i = 0), this equation can be solved on *E* at fixed real **k**, which is reduced to the standard eigenvalue

problem for a Hermitian matrix with the elements $\left\{ \frac{\hbar^2}{2m} |\mathbf{k} + \mathbf{G}|^2 \delta_{\mathbf{GG'}} + V_{\mathbf{GG'}} \right\}$ (подразумевается $V_i = 0$). For complex **k**, however, $E(\mathbf{k})$ is in general complex even with $V_i = 0$, and stays the physically meaningful real energy only along so-called 'real lines' as a function of **k**. Therefore, one has to solve the secular equation backwards, i.e. find **k** at fixed *E*, which is a laborious non-linear problem.

If ***V*** does not depend on $k_\perp$ [14,16] (which is the case for local ***V***) or depends on $k_\perp$ linearly as

$$\{V_{\mathbf{GG'}}(k_\perp)\} = \{V^0_{\mathbf{GG'}}\} + k_\perp \{V^1_{\mathbf{GG'}}\},$$

the secular equation on complex $k_\perp$ reduces to the generalized eigenvalue problem

$$\begin{Bmatrix} -\boldsymbol{P}^- & -\boldsymbol{Q} - \{V^0_{\mathbf{GG'}}\} \\ \boldsymbol{I} & -\boldsymbol{P}^+ \end{Bmatrix} \mathbf{X} - k_\perp \begin{Bmatrix} \boldsymbol{I} & \{V^1_{\mathbf{GG'}}\} \\ 0 & \boldsymbol{I} \end{Bmatrix} \mathbf{X} = 0$$

for the matrix $\begin{Bmatrix} -\boldsymbol{P}^- & -\boldsymbol{Q} - \{V^0_{\mathbf{GG'}}\} \\ \boldsymbol{I} & -\boldsymbol{P}^+ \end{Bmatrix}$, where $\boldsymbol{I} = \{\delta_{\mathbf{GG'}}\}$, $\boldsymbol{P}^\pm = \left\{ \left(\frac{\hbar}{\sqrt{2m}} G_\perp \pm \sqrt{E} \right) \delta_{\mathbf{GG'}} \right\}$ and $\boldsymbol{Q} = \left\{ \frac{\hbar^2}{2m} |\mathbf{k}_{//} + \mathbf{G}_{//}|^2 \delta_{\mathbf{GG'}} - iV_i \delta_{\mathbf{GG'}} \right\}$, with the eigenvector $\mathbf{X} = \begin{Bmatrix} (\boldsymbol{P}^+ + k_\perp \boldsymbol{I})\{C_{\mathbf{GG'}}\} \\ \{C_{\mathbf{GG'}}\} \end{Bmatrix}$ (for details see Ref. 6). Although this matrix is quite awkward (double dimensioned, complex and non-Hermitian) such a reduction of the non-linear secular equation to a standard linear-algebra problem is an immense reduction of the computational complexity.

The above computational scheme has been used to calculate the complex band structure in Fig. 4 of the main article. The calculations in Fig. 4 (b) are represented in Supplementary Figure 3 fully as a function of both Re$k_\perp$ and Im$k_\perp$.

Using local linearization of $V_{\mathbf{GG'}}(k_\perp)$, this method can in principle be extended to the non-local pseudopotentials in first-principles calculations. Complex $E(\mathbf{k})$ can also be calculated based on other first-principles calculations (for example, FLAPW) using **k·p** expansion [16,18]. In this case the representation $\phi_\mathbf{k} = \sum_n C_n u_n(\mathbf{r}) \, exp(i\mathbf{kr})$ is used, where $u_n(\mathbf{r})$ are the set of $\phi_\mathbf{k}$ for a fixed real $k_\perp$ normally chosen in the middle of the interval of interest. In this case, the secular equation is also reduced to the linear eigenvalue problem for a double-dimensioned non-Hermitian matrix.

2.2 Wavefunction-matching description of the LEED process

Having calculated complex $E(\mathbf{k})$ for the direction $\mathbf{k}_{//} = \mathbf{K}_{//} + \mathbf{g}$ in the Brillouin zone defined by the parallel-momentum conservation, the amplitudes of the plane waves diffracted off the crystal and of the Bloch waves excited in the crystal can be calculated by matching them at the crystal surface [12-14,19]. Within this formalism, the wavefunction on the vacuum side is represented as a superposition of the primary plane wave and all diffracted beams corresponding to the surface reciprocal vectors **g** as

$$\Phi_{\text{vac}}(\mathbf{r}) = A exp(i\mathbf{Kr}) + \sum_\mathbf{g} R_\mathbf{g} \, exp(-i\mathbf{K}_\mathbf{g}\mathbf{r})$$

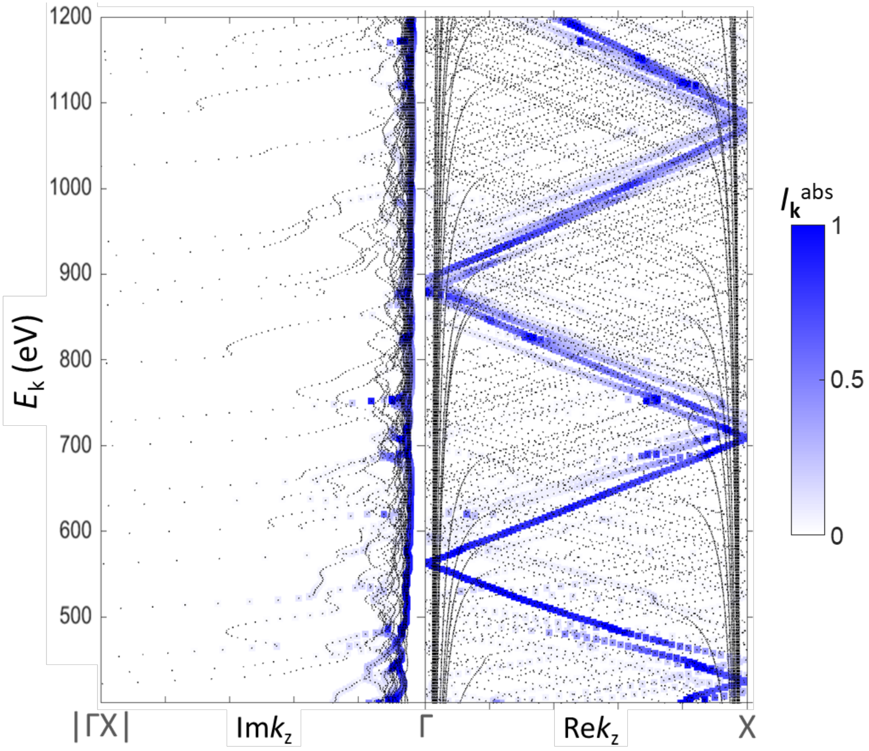

Supplementary Figure 3. Complex band structure of the model fcc crystal from Fig. 4(b), which includes its dependence on Im$k_\perp$ representing damping of the Bloch waves into the crystal interior. The partial currents $I_{\mathbf{k}}^{abs}$, shown in blue scale, identify the activity of the bands in both LEED and PE processes.

where $|A| = (2K_\perp)^{-1/2}$ normalises the primary current to unity, and each $\mathbf{K}_{\mathbf{g}//}$ is defined by the parallel-momentum conservation as $\mathbf{K}_{\mathbf{g}//} = \mathbf{K}_{//} + \mathbf{g}$ and $K_{\mathbf{g}\perp}$ by the energy conservation as $K_{\mathbf{g}\perp} = \sqrt{K^2 - \left(K_{\mathbf{g}//}\right)^2}$. Importantly, the superposition includes the propagating beams with real $K_{\mathbf{g}\perp}$ as well as the ones exponentially decaying in vacuum with complex $K_{\mathbf{g}\perp}$. In turn, the wavefunction on the crystal side is represented as a superposition of Bloch waves $\phi_{\mathbf{k}}$

$$\Phi_c(\mathbf{r}) = \sum_{\mathbf{k}} T_{\mathbf{k}} \phi_{\mathbf{k}}(\mathbf{r}),$$

where each $\mathbf{k}_{//}$ is again defined by the parallel-momentum conservation as $\mathbf{k}_{//} = \mathbf{K}_{//} + \mathbf{g}$ and complex $k_\perp$ by energy conservation with the complex $E(k_\perp)$. Importantly, the superposition includes bulk-derived $\phi_{\mathbf{k}}$ which are forced to decay into the crystal by V_i only, and the surface-derived ones which decay more rapidly because of the elastic scattering off the crystal potential. The latter include so-called gap states in the band gaps of the infinite-crystal band structure [13,15,16].

The amplitudes $R_{\mathbf{g}}$ and $T_{\mathbf{k}}$ are then determined by matching $\Phi_{vac}(\mathbf{r})$ and $\Phi_c(\mathbf{r})$ (equating their amplitudes and derivatives) at the matching plane $r_\perp = r_\perp^0$. The latter is normally chosen at half the interlayer distance from the top atomic plane, although the results are not very sensitive to its exact position. For the matching, $\Phi_{vac}(\mathbf{r})$ and $\Phi_c(\mathbf{r})$ are expanded over $N_{\mathbf{g}}$ surface-parallel plane waves $exp(i(\mathbf{K}_{//} + \mathbf{g})\mathbf{r}_{//})$ [12] (for an alternative matrix approach see [13,17]). The former expands as

$$\Phi_{vac}(\mathbf{r}) = \sum_{\mathbf{g}} \left(\delta_{\mathbf{g},0} A exp(iK_{\mathbf{g}\perp} r_\perp) + R_{\mathbf{g}}\, exp(-iK_{\mathbf{g}\perp} r_\perp)\right) exp(i(\mathbf{K}_{//} + \mathbf{g})\mathbf{r}_{//}),$$

where the primary wave is represented as $\sum_{\mathbf{g}} \delta_{\mathbf{g},0} A exp\left(iK_{\mathbf{g}\perp} r_\perp\right) exp\left(i(\mathbf{K}_{//} + \mathbf{g})\mathbf{r}_{//}\right)$. The latter, in turn, expands as

$$\Phi_{\mathrm{c}}(\mathbf{r}) = \sum_{\mathbf{g}} \left(\sum_{\mathbf{k}} T_{\mathbf{k}} C_{\mathbf{kg}}(r_\perp) \right) exp\left(i(\mathbf{K}_{//} + \mathbf{g})\mathbf{r}_{//}\right),$$

where each $\phi_{\mathbf{k}}$ are represented as the two-dimensional Laue expansion over **g** as $\phi(\mathbf{r}) = \sum_{\mathbf{g}} C_{\mathbf{kg}}(r_\perp) exp\left(i(\mathbf{K}_{//} + \mathbf{g})\mathbf{r}_{//}\right)$. Here, the coefficients $C_{\mathbf{kg}}(r_\perp)$ are defined as $\int \phi(\mathbf{r}) exp\left(-i(\mathbf{K}_{//} + \mathbf{g})\mathbf{r}_{//}\right) d\mathbf{r}_{//}$, which can be found from the three-dimensional Fourier expansion $\phi_{\mathbf{k}} = \sum_{\mathbf{G}} C_{\mathbf{G}} e^{i(\mathbf{k}+\mathbf{G})\mathbf{r}}$ as $C_{\mathbf{kg}}(r_\perp) = \sum_{\mathbf{G}} \delta_{\mathbf{g},\mathbf{G}_{//}} C_{\mathbf{G}} e^{i(k_\perp + G_\perp) r_\perp}$. Then the matching of $\Phi_{vac}(\mathbf{r})$ и $\Phi_{c}(\mathbf{r})$ is equivalent to matching of each Laue-expansion coefficient as

$$\begin{cases} \delta_{\mathbf{g},0} A exp\left(iK_{\mathbf{g}\perp} r_\perp^0\right) + R_{\mathbf{g}}\, exp\left(-iK_{\mathbf{g}\perp} r_\perp^0\right) = \sum_{\mathbf{k}} T_{\mathbf{k}} C_{\mathbf{kg}}\left(r_\perp^0\right) \\ iK_{\mathbf{g}\perp}\left(\delta_{\mathbf{g},0} A exp\left(iK_{\mathbf{g}\perp} r_\perp^0\right) - R_{\mathbf{g}}\, exp\left(-iK_{\mathbf{g}\perp} r_\perp^0\right)\right) = \sum_{\mathbf{k}} T_{\mathbf{k}} \left[\frac{\partial}{\partial r_\perp} C_{\mathbf{kg}}(r_\perp) \right]_{r_\perp = r_\perp^0} \end{cases},$$

which forms an inhomogeneous system of $2N_{\mathbf{g}}$ linear equation (so-called matching system) on $R_{\mathbf{g}}$ и $T_{\mathbf{k}}$. Importantly, this system is determined only if the number of included $\phi_{\mathbf{k}}$ is equal to $N_{\mathbf{g}}$. In general, this condition forces inclusion of a large number of rapidly decaying $\phi_{\mathbf{k}}$ which may reduce the numerical stability of the matching calculations [18,20]. The surface effects can be included into the matching formalism by embedding an additional region between the vacuum and the crystal, with the potential in this region describing the surface barrier and possible surface reconstructions, and matching the wavefunctions on the two planes on both sides of the embedded region [14,20].

2.3 Partial absorbed currents and the current conservation

In the $V_i = 0$ limit, the current carried into the crystal in the LEED process is defined as the sum $\sum_{\mathbf{k}} |T_{\mathbf{k}}|^2 \upsilon_{\mathbf{gk}\perp}$ over all propagating $\phi_{\mathbf{k}}$, where $\upsilon_{\mathbf{gk}\perp}$ is their surface-perpendicular group velocity. When the electron absorption is introduced through $V_i \neq 0$, all $\phi_{\mathbf{k}}$ become decaying, and can not carry current in its conventional sense. It has been realized, however, that within this formalism the physical origin of the current carried into the crystal in the LEED process is exactly the inelastic processes described by $V_i \neq 0$ and causing $\phi_{\mathbf{k}}$ to decay [9] because the electrons washed away from the coherent wavefunction via V_i undergo cascade transitions towards the Fermi level and carry current into the crystal via the conventional electron transport mechanism. Then the total current I^{abs} absorbed in the LEED process is proportional to the rate of electron absorption in time, i.e. to the dissipation rate of the coherent wavefunction in the crystal

$$I^{\mathrm{abs}} = -\frac{d}{dt} \int \left|\Phi_{\mathrm{c}}(\mathbf{r}, t)\right|^2 d\mathbf{r}$$

(assuming that $\Phi_c(\mathbf{r},t)$ is normalized to the surface area and the integration runs over the whole semi-infinite crystal). Inserting into this expression $\Phi_c(\mathbf{r},t)$ in its explicit time-dependent represenation

$\Phi_c(\mathbf{r},t) = \Phi_c(\mathbf{r})\, exp(-iEt/\hbar)\, exp(-t/\tau)$, where τ is the electron lifetime determining $V_i = \hbar/\tau$, we arrive at the stationary expression

$$I^{abs} = V_i \int \left|\Phi_c(\mathbf{r})\right|^2 d\mathbf{r} ,$$

where $V_i = 1/\tau$ and the time-decay term $exp(-2t/\tau)$ before the integral is set to 1 (physically, the time decay is refilled by the incident electrons). In words, the total current absorbed in the LEED process is sheer the integral coherent electron density in the crystal multiplied by the electron absorption rate. Furthermore, the Bloch-wave expansion $\Phi_c = \sum_{\mathbf{k}} T_{\mathbf{k}} \phi_{\mathbf{k}}$ separates I^{abs} into partial absorbed currents for each $\phi_{\mathbf{k}}$

$$I_{\mathbf{k}}^{abs} = V_i \int \left| T_{\mathbf{k}} \phi_{\mathbf{k}}(\mathbf{r})\right|^2 d\mathbf{r} ,$$

provided that the cross-terms $\int \phi_{\mathbf{k}'}^*(\mathbf{r}) \phi_{\mathbf{k}}(\mathbf{r}) d\mathbf{r}$ stay are enough (in other words, $\phi_{\mathbf{k}}$ and $\phi_{\mathbf{k}'}$ close to orthogonality). These $I_{\mathbf{k}}^{abs}$, proportional to the integral coherent electron density in each $\phi_{\mathbf{k}}$ (and the corresponding energy band) determine their activity in the LEED process.

This formalism, developed in [9], directly relates the LEED process to particular bands in the whole multitude of unoccupied electronic bands. A vivid example is the calculations in Fig. 4, where the $I_{\mathbf{k}}^{abs}$ values are represented in blue scale. Out of the massive number of bands folded back to the reduced Brillouin zone, the large $I_{\mathbf{k}}^{abs}$ confidently identify the few active ones.

Stunningly, for $V_i \to 0$ the above expression for $I_{\mathbf{k}}^{abs}$ reduces to the usual current $I_{\mathbf{k}}^{abs} = \left|T_{\mathbf{k}}\right|^2 \upsilon_{g\perp}$ carried by the propagating Bloch wave. Indeed, the Fourier expansion $\phi_{\mathbf{k}} = \sum_{\mathbf{G}} C_{\mathbf{G}}^{\mathbf{k}}\, exp(i(\mathbf{k}+\mathbf{G})\mathbf{r}) = \sum_{\mathbf{G}} exp(-i\, Im k_\perp r_\perp) C_{\mathbf{G}}^{\mathbf{k}}\, exp(i(\mathbf{k}+\mathbf{G})\mathbf{r})$ transforms $I_{\mathbf{k}}^{abs}$ as

$$I_{\mathbf{k}}^{abs} = V_i \int \left| T_{\mathbf{k}} \phi_{\mathbf{k}}(\mathbf{r})\right|^2 d\mathbf{r} =$$

$$V_i \int \left|T_{\mathbf{k}}\right|^2 \left(exp(-i\, Im k_\perp r_\perp) \cdot \sum_{\mathbf{G},\mathbf{G}'} C_{\mathbf{G}}^{\mathbf{k}*} C_{\mathbf{G}'}^{\mathbf{k}}\, exp(i(\mathbf{G}-\mathbf{G}')\mathbf{r}) \right) d\mathbf{r} =$$

$$V_i \left|T_{\mathbf{k}}\right|^2 \sum_{\mathbf{G},\mathbf{G}'} C_{\mathbf{G}}^{\mathbf{k}*} C_{\mathbf{G}'}^{\mathbf{k}} \int exp(-i\, Im k_\perp r_\perp) \cdot exp(i(\mathbf{G}-\mathbf{G}')\mathbf{r}) d\mathbf{r}$$

Separating **G** into the parallel and perpendicular components and using the orthogonality relation $\int exp(i(\mathbf{G}_{//} - \mathbf{G}'_{//})\mathbf{r}) d\mathbf{r}_{//} = \delta_{\mathbf{G}_{//}-\mathbf{G}'_{//}}$, we arrive at

$$I_{\mathbf{k}}^{abs} = V_i \left|T_{\mathbf{k}}\right|^2 \sum_{\mathbf{G},\mathbf{G}'} \delta_{\mathbf{G}_{//}-\mathbf{G}'_{//}} C_{\mathbf{G}}^{\mathbf{k}*} C_{\mathbf{G}'}^{\mathbf{k}} \frac{i}{\left|G_\perp - G'_\perp\right| + 2i\, Im\, k_\perp}$$

For the bulk-derived $\phi_{\mathbf{k}}$, which are the only ones to carry current in the $V_{\mathrm{i}} \rightarrow 0$ limit, $Im k_{\perp} \rightarrow 0$. Then $\frac{i}{\left|G_{\perp}-G_{\perp}^{'}\right|+2i\,Im\,k_{\perp}} \rightarrow \frac{1}{2\,Im\,k_{\perp}}\delta_{G_{\perp}-G_{\perp}^{'}}$, and we obtain

$$I_{\mathbf{k}}^{\mathrm{abs}} = V_{\mathrm{i}}\left|T_{\mathbf{k}}\right|^{2}\frac{1}{2\,Im\,k_{\perp}}\sum_{\mathbf{G},\mathbf{G}'}\delta_{\mathbf{G}_{//}-\mathbf{G}_{//}^{'}}\,\delta_{G_{\perp}-G_{\perp}^{'}}\,C_{\mathbf{G}}^{\mathbf{k}*}C_{\mathbf{G}'}^{\mathbf{k}} =$$

$$V_{\mathrm{i}}\left|T_{\mathbf{k}}\right|^{2}\frac{1}{Im\,k_{\perp}}\sum_{\mathbf{G}}\left|C_{\mathbf{G}}^{\mathbf{k}}\right|^{2}$$

With the completeness relation $\sum_{\mathbf{G}}\left|C_{\mathbf{G}}^{\mathbf{k}}\right|^{2}=1$ and $Im\,k_{\perp} = \frac{V_{\mathrm{i}}}{\upsilon_{\mathrm{g}\perp}}$ for the bulk $\phi_{\mathbf{k}}$, we finally arrive at

$$I_{\mathbf{k}}^{\mathrm{abs}} = \left|T_{\mathbf{k}}\right|^{2}\upsilon_{\mathrm{g}\perp}$$

which is the conventional expression for the current carried by the propagating Bloch wave.

The main factor affecting the accuracy of the matching calculations is a truncation of the basis set, violating the completeness of the **k**-space representation of $\phi_{\mathbf{k}}$. The current conservation, generalized to $V_{\mathrm{i}} \neq 0$ as the conservation of the total $I^{\mathrm{abs}} = V_{\mathrm{i}}\int\left|\Phi_{\mathrm{c}}(\mathbf{r})\right|^{2}d\mathbf{r}$ absorbed in the crystal, appears then as the most vivid criterion controlling of the computational accuracy [9-11]. We note that I^{abs} should embrace all partial $I_{\mathbf{k}}^{\mathrm{abs}}$ as well as the cross-terms $\int\phi_{\mathbf{k}'}^{*}(\mathbf{r})\phi_{\mathbf{k}}(\mathbf{r})d\mathbf{r}$.

2.4 Partial contributions of the Bloch waves in PE vs LEED

Within the one-step theory of photoemission (PE) [21,22], the photocurrent I^{ph} is described as the matrix element of the operator $\mathbf{A}\cdot\mathbf{p}$, where $\mathbf{A}$ is the vector potential of the electromagnetic field and $\mathbf{p}$ the momentum operator, between the one-electron initial state Φ^{i} and final state Φ^{f}

$$I^{\mathrm{ph}} \propto \left|\left\langle\Phi^{\mathrm{f}*}(\mathbf{r})\middle|\mathbf{A}\cdot\mathbf{p}\middle|\Phi^{\mathrm{i}}(\mathbf{r})\right\rangle\right|^{2}$$

Φ^{f} being identical to the time-reversed LEED state sets up a direct connection between the PE and LEED processes. In order to extend this connection to the individual Bloch-wave components $\phi^{\mathrm{f}}_{\mathbf{k}}$ [12] of the total Φ^{f}, the latter can be expanded as $\sum_{\mathbf{k}}T_{\mathbf{k}}\phi_{\mathbf{k}}^{\mathrm{f}}(\mathbf{r})$, where $T_{\mathbf{k}}$ are the excitation amplitudes of $\phi^{\mathrm{f}}_{\mathbf{k}}$ in the LEED process. For brevity, let Φ^{i} include one single Bloch wave ϕ^{i}. Then the above I^{ph} can be represented as

$$I^{\mathrm{ph}} \propto \sum_{\mathbf{k}}\left|T_{\mathbf{k}}\right|^{2}\left|\left\langle\phi_{\mathbf{k}}^{\mathrm{f}*}(\mathbf{r})\middle|\mathbf{A}\cdot\mathbf{p}\middle|\phi^{\mathrm{i}}(\mathbf{r})\right\rangle\right|^{2} + \sum_{\mathbf{k}\neq\mathbf{k}'}T_{\mathbf{k}}^{*}T_{\mathbf{k}'}^{*}\left\langle\phi_{\mathbf{k}}^{\mathrm{f}*}(\mathbf{r})\middle|\mathbf{A}\cdot\mathbf{p}\middle|\phi^{\mathrm{i}}(\mathbf{r})\right\rangle\left\langle\phi_{\mathbf{k}'}^{\mathrm{f}*}(\mathbf{r})\middle|\mathbf{A}\cdot\mathbf{p}\middle|\phi^{\mathrm{i}}(\mathbf{r})\right\rangle$$

With moderate values of V_{i} the cross-terms at $\mathbf{k} \neq \mathbf{k}'$ stay relatively small. In analogy with the total I^{abs} and partial $I_{\mathbf{k}}^{\mathrm{abs}}$ in the LEED process, I^{ph} can be separated into the partial photocurrents $I_{\mathbf{k}}^{\mathrm{ph}}$ from each $\phi_{\mathbf{k}}^{\mathrm{f}}$,

$$I_{\mathbf{k}}^{\mathrm{ph}} \propto \left|T_{\mathbf{k}}\right|^{2}\left|\left\langle\phi_{\mathbf{k}}^{\mathrm{f}*}(\mathbf{r})\middle|\mathbf{A}\cdot\mathbf{p}\middle|\phi^{\mathrm{i}}(\mathbf{r})\right\rangle\right|^{2}.$$

As $\left|T_{\mathbf{k}}\right|^2$ in the LEED process is related to $I_{\mathbf{k}}^{\mathrm{abs}}$ as $\left|T_{\mathbf{k}}\right|^2 \propto \frac{1}{V_{\mathrm{i}}} I_{\mathbf{k}}^{\mathrm{abs}}$, we arrive at

$$I_{\mathbf{k}}^{\mathrm{ph}} \propto \left(\frac{1}{V_{\mathrm{i}}} \left| \left\langle \phi_{\mathbf{k}}^{\mathrm{f}*}(\mathbf{r}) \middle| \mathbf{A} \cdot \mathbf{p} \middle| \phi^{\mathrm{i}}(\mathbf{r}) \right\rangle \right|^2 \right) \cdot I_{\mathbf{k}}^{\mathrm{abs}}$$

This expression demonstrates that the partial $I_{\mathbf{k}}^{\mathrm{ph}}$ from the individual final-state $\phi_{\mathbf{k}}^{\mathrm{f}}$ in the PE process are proportional to the partial $I_{\mathbf{k}}^{\mathrm{abs}}$ in the LEED process. Therefore, analysis of $I_{\mathbf{k}}^{\mathrm{abs}}$ such as shown in Supplementary Figure 3 confidently identifies the bands active in both processes.

Summarizing, the matching approach of LEED for the semi-infinite crystal traces the most elegant method to connect the LEED and ARPES spectra to the individual Bloch waves (and corresponding bands) constituting the PE final state. Because of the underlying complex band structure calculations, this method is computationally expensive, however. Alternative methods based on multiple scattering, discussed above, are an order of magnitude more efficient numerically, but their connection to the final-state bandstructure is more obscure because they generate the total final-state wavefield without its decomposition into the individual Bloch waves.

Supplementary References

1. S. H. Vosko, L. Wilk, and M. Nusair, Accurate Spin-Dependent Electron Liquid Correlation Energies for Local Spin Density Calculations: A Critical Analysis, Canadian J. Phys. 58 (1980) 1200
2. H. Ebert, D. Ködderitzsch, and J. Minár, Calculating Condensed Matter Properties Using the KKR-Green's Function Method – Recent Developments and Applications, Rep. Prog. Phys. 74 (2011) 096501.
3. J. M. MacLaren, S. Crampin, D. D. Vvedensky, and J. B. Pendry, Layer Korringa-Kohn-Rostoker Technique for Surface and Interface Electronic Properties, Phys. Rev. B 40 (1989) 12164
4. J. Braun, J. Minár, and H. Ebert, Correlation, Temperature and Disorder: Recent Developments in the One-Step Description of Angle-Resolved Photoemission, Phys. Rep. 740 (2018) 1.
5. Multiple Scattering Theory for Spectroscopies, eds. D. Sébilleau, K. Hatada and H. Ebert. Springer Proc. Phys. 204 (2018).
6. J. Braun, The theory of angle-resolved ultraviolet photoemission and its applications to ordered materials. Rep. Prog. Phys. 59 (1996) 1267.
7. J. Braun, J. Minár, S. Mankovsky, V. N. Strocov, N. B. Brookes, L. Plucinski, C. M. Schneider, C. S. Fadley, and H. Ebert, Exploring the XPS Limit in Soft and Hard X-Ray Angle-Resolved Photoemission Using a Temperature-Dependent One-Step Theory, Phys. Rev. B 88 (2013) 205409
8. D. Sébilleau, S. Tricot, and A. Koide, unpublished (2022).
9. V. N. Strocov, H. Starnberg & P. O. Nilsson. Excited-state bands of Cu determined by VLEED band fitting & their implications for photoemission, Phys. Rev. B 56 (1997) 1717.
10. V. N. Strocov, R. Claessen, G. Nicolay, S Hüfner, A Kimura, A. Harasawa, S. Shin, A. Kakizaki, P.O. Nilsson, H.I. Starnberg & P. Blaha. Three-dimensional band mapping by angle-dependent very-low-energy electron diffraction and photoemission: Methodology and application to Cu. Phys. Rev. B 63 (2001) 20510.
11. V. N. Strocov, E.E. Krasovskii, W. Schattke, N. Barrett, H. Berger, D. Schrupp & R. Claessen. Three-dimensional band structure of layered $TiTe_2$: Photoemission final-state effects. Phys. Rev. B 74 (2006) 195125
12. G. Capart. Band structure calculations of low energy electron diffraction at crystal surfaces. Surf. Sci. **13** (1969) 361
13. J. B. Pendry. *Low-Energy Electron Diffraction* (Academic Press, London, 1974)

14. J. B. Pendry. The application of pseudopotentials to low-energy electron diffraction II: Calculation of the reflected intensities. J. Phys. C **2** (1969) 2273
15. H. Bross. Methods to evaluate Heine's complex band structure. Surf. Sci. **213** (1989) 215
16. D. L. Smith, C. Mailhiot. Theory of semiconductor superlattice electronic structure. Rev. of Mod. Phys. **62** (1990) 173
17. J. B. Pendry. Ion core scattering and low-energy electron diffraction - II. J. Phys.C **4** (1971) 2514
18. E. E. Krasovskii, W. Schattke. Surface electronic structure with the linear methods of band theory. Phys. Rev. B **56** (1997) 12874
19. R. C. Jaklevic, L. C. Davis. Band structure signatures in the low-energy electron reflectance spectra of fcc metals. Phys. Rev. B **26** (1982) 5391
20. E. E. Krasovskii, W. Schattke. Calculation of the wave functions for semi-infinite crystals with linear methods of band theory. Phys. Rev. B **59** (1999) R15 609
21. P. J. Feibelman, D. E. Eastman. Photoemission spectroscopy - Correspondence between quantum theory and experimental phenomenology. Phys. Rev. B **10** (1974) 4932
22. J. B. Pendry. Theory of photoemission. Surf. Sci. **57** (1976) 679
